# Expanding Omics Resources for Improvement of Soybean Seed Composition Traits

**DOI:** 10.3389/fpls.2015.01021

**Published:** 2015-11-24

**Authors:** Juhi Chaudhary, Gunvant B. Patil, Humira Sonah, Rupesh K. Deshmukh, Tri D. Vuong, Babu Valliyodan, Henry T. Nguyen

**Affiliations:** Division of Plant Sciences, National Center for Soybean Biotechnology, University of MissouriColumbia, MO, USA

**Keywords:** legumes, soybean, seed traits, omics, genomics, next-generation sequencing (NGS), quantitative trait loci (QTL), genome-wide association study (GWAS)

## Abstract

Food resources of the modern world are strained due to the increasing population. There is an urgent need for innovative methods and approaches to augment food production. Legume seeds are major resources of human food and animal feed with their unique nutrient compositions including oil, protein, carbohydrates, and other beneficial nutrients. Recent advances in next-generation sequencing (NGS) together with “omics” technologies have considerably strengthened soybean research. The availability of well annotated soybean genome sequence along with hundreds of identified quantitative trait loci (QTL) associated with different seed traits can be used for gene discovery and molecular marker development for breeding applications. Despite the remarkable progress in these technologies, the analysis and mining of existing seed genomics data are still challenging due to the complexity of genetic inheritance, metabolic partitioning, and developmental regulations. Integration of “omics tools” is an effective strategy to discover key regulators of various seed traits. In this review, recent advances in “omics” approaches and their use in soybean seed trait investigations are presented along with the available databases and technological platforms and their applicability in the improvement of soybean. This article also highlights the use of modern breeding approaches, such as genome-wide association studies (GWAS), genomic selection (GS), and marker-assisted recurrent selection (MARS) for developing superior cultivars. A catalog of available important resources for major seed composition traits, such as seed oil, protein, carbohydrates, and yield traits are provided to improve the knowledge base and future utilization of this information in the soybean crop improvement programs.

## Introduction

In view of the increasing world population, production of sustainable food supplies will be a critical challenge in the twenty-first century. The world population is projected to cross 9 billion by 2050, indicating that food supplies must be doubled to meet the requirement of the expanding population (Varshney et al., 2013a; Zhou et al., 2015). Apart from the quantity of food, quality is also a critical issue to maintain nutritive values with increased potential for yield. Seeds are an important part of the plant due to their role in reproduction and storing food reserves in the embryonic cotyledons. Legume seeds are an essential source of food, feed, minerals, and also provides biological nitrogen fixation by forming a symbiotic relationship with rhizobia (Gepts et al., 2005). Soybeans are unique in legumes with a seed content of about 40% protein and 21% oil on a dry matter basis. It is the most widely grown oil seed crop in the world and represented 56% of the world's vegetable oil seed production in 2013. The United States is the leading soybean producer with 34% [108 Million Metric Tons (MMT)], followed by Brazil with 30% (94.5 MMT), and Argentina with 18% (56 MMT) of the world production (SoyStats 2014, www.soystats.com). Besides the total seed oil and protein content, oil components (fatty acids) and protein components (amino acids) are also desirable for long term shelf life and nutrition. The animal feed industry uses about 70% of soybean meal due to it being high in protein with a good amino acid balance. Soybean meal provides more energy than any other plant protein source (Cromwell, 2012). Furthermore, soybean is also used as sources of industrial and pharmaceutical applications as well as in the production of biodiesel (Goldberg and Stacey, 2008). Several international and domestic soybean processors prefer soybean with different combinations of seed composition. Soybean fatty acids include palmitic acid (10%), stearic acid (4%), oleic acid (18%), linoleic acid (55%), and linolenic acid (13%). Higher oleic acid and lower linolenic acid without generating *trans*-fats are desirable for oil stability and addressing health concerns of soybean oil (Clemente and Cahoon, 2009; Lee et al., 2012). In addition, manipulation of the amino acid profile [methionine (Met), lysine (Lys), and threonine (Thr)], is desired to improve seed protein quality since the animal feed industry uses about 77% of soybean meal as a source of protein and amino acids (Warrington et al., 2015).

Another important component of soybean seeds are carbohydrates. Their concentration is very crucial for determining animal feed quality (Wilson, 2004). Soybean seeds contain about 12% soluble carbohydrates, which includes sucrose, raffinose, and stachyose. Among the three carbohydrates, only higher sucrose content is desirable as metabolizable energy in the animal feed. Raffinose and stachyose accumulation causes indigestibility and flatulence which ultimately results in reduced economic and dietary value of soybean seed (Hagely et al., 2013). Therefore, it is a prerequisite to improve seed utility by adjusting the concentration of the above-mentioned components in a desired proportion.

**Figure 1:**
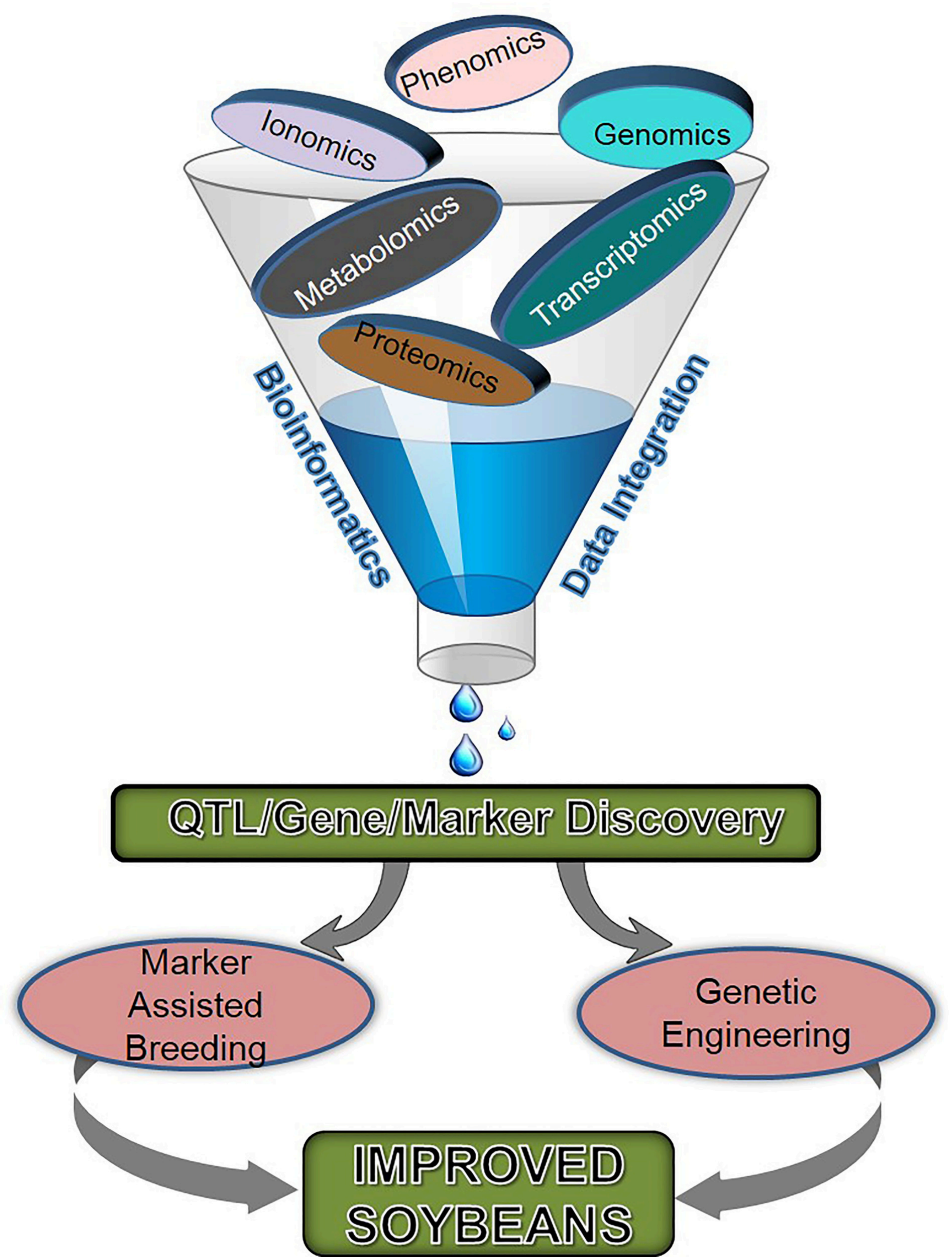
**Different omics approaches and their integrated tools being used for soybean breeding**.

The soybean seed composition traits are considered complex due to multiple gene control, environmental factors, and the interaction between molecular, biochemical, and genetic mechanisms within the seed. The negative correlation of protein with oil and yield is poorly understood and breaking this relationship is another challenge in soybean improvement (Clemente and Cahoon, 2009). Additionally, the variation in seed composition is largely affected by environment; for example, cooler temperature negatively affects seed storage protein content (Bellaloui et al., 2015). Therefore, the identification of environmentally stable germplasm with desirable seed composition and elucidating their genetic control and complex metabolic interactions during seed development are needed for soybean improvement. Such a multi-tier investigation requires extensive experimental efforts, which involves genomic, transcriptomic, metabolomic, ionomic, and phenomic tools (Figure 1).

The development of soybean seed with high quality and improved yield can be accelerated using modern breeding techniques, such as marker-assisted selection (MAS) (Xu and Crouch, 2008), genomic selection (Desta and Ortiz, 2014), and genome editing approaches (Voytas and Gao, 2014). In recent years, transcriptome analysis (Severin et al., 2010), proteomics (Eldakak et al., 2013), metabolomics (Saito and Matsuda, 2010) phenomics (Zhu et al., 2012), and ionomics (Singh et al., 2013b) have progressed at a rapid pace. A comprehensive overview summarizing increasing research efforts in soybean genomics, transcriptomics, and proteomics during the last decade is highlighted in the Supplementary Figure 1.

The soybean genome was the first published legume reference genome (Schmutz et al., 2010) and was followed by other legume genome sequences (Varshney et al., 2012a, 2013b; Schmutz et al., 2014). Until recently, with the advancement in next-generation sequencing (NGS) technology, whole genome sequencing (WGS) has become possible for all major crops, including soybean and is also being utilized for several orphan legume species (Varshney et al., 2012b). Due to low sequencing cost, NGS has been widely used in various *de novo* sequencing, whole genome re-sequencing (WGRS), genotyping-by-sequencing (GBS), and transcriptome analysis. This has made a significant impact in molecular breeding programs through marker development and agronomic traits mapping (Metzker, 2010; Peterson et al., 2012; Poland and Rife, 2012; Varshney et al., 2012b; Sonah et al., 2013).

Although rapid progresses in the use of omics tools have been demonstrated, data mining and analyses are still challenging tasks. There is a wide range of genetic variation in oil and protein content among soybean accessions of the USDA Soybean Germplasm Collection, but it is extremely rare to find an accession with higher protein and oil content (Wilson, 2004). For decades, geneticists have used a quantitative trait loci (QTL) mapping approach to identify major genes responsible for seed composition traits, yielding several putative candidate genes, but currently, there are no precise genomic loci identified for these traits in soybean. Technological advances in sensitivity, resolution, high-throughput, and reduced costs of the “omics” based assays have provided a doorway for the applications of complex trait studies. The resulting data includes molecular markers, transcript sequences, genetic linkage maps, and physical maps; all of which would help in the elucidation of complex traits. Therefore, the integration of several “omics” platforms can be an excellent approach for the assessment of various seed composition traits. This review aims to highlight significant studies using omics approaches such as genomics, transcriptomics, metabolomics, proteomics, and phenomics applied to soybean seed composition improvement.

## Genomics development

### Molecular mapping of seed composition traits in soybean

Molecular markers allow precise, cost effective, and high-throughput identification of genetic variants for different traits. Markers are important in breeding applications for developing genetic linkage maps, germplasm evaluation, phylogenetic and evolutionary analysis, selection of desired alleles and mapping of genes/QTL. Simple sequence repeat (SSR) markers have been extensively utilized to study seed composition traits in soybean (Wang et al., 2014b; Warrington et al., 2015); for example, seed oil, protein, and seed size QTL (Hyten et al., 2004), fine-mapping of soybean protein QTL on chromosome (Chr.) 20 (Nichols et al., 2006). A publicly available SSR marker database, containing about 33,000 markers was developed from WGS information (Song et al., 2010). Eskandari et al. (2013) utilized SSR markers and identified QTL for oil content on Chr. 9, which also had a significant positive effect on seed protein composition. For the improvement of soybean meal, Pathan et al. (2013) detected QTL using both SSR and single nucleotide polymorphism (SNP) markers for seed protein, oil, and seed weight across genetic backgrounds and environments on Chrs. 5 and 6. The SSR markers are less abundant in the genome and has limitations in high-throughput applicability as compared to SNP markers to be utilized in large breeding programs (Singh et al., 2013a).

The availability of a well-annotated soybean genome sequence has facilitated the development of SNP markers and is being utilized in crop improvement (Table 1). The genotyping approaches include GBS (Elshire et al., 2011; Sonah et al., 2013), restriction site associated DNA sequencing (Baird et al., 2008), SoySNP50K iSelect BeadChip (Song et al., 2013), SoySNP6K Infinium BeadChip (Akond et al., 2013), and the Axiom SoyaSNP array for approximately 180,000 SNPs (Lee et al., 2015). Furthermore, NGS has also facilitated the development of various SNP genotyping assays, such as KASPar, GoldenGate, and Infinium Chips, which can also be applied in genome-wide marker development (Varshney et al., 2015).

**Table 1:** **Whole genome re-sequencing efforts performed in soybean**.

**Sr. No**.	**No. of lines used**	**Genotypes**	**Sequencing depth**	**SNP calling method**	**No. of SNPs**	**References**
1	1	*G. soja*	~52.07X	De novo	~2.5 Million	Kim et al., 2010
2	31	17 *G. soja* and 14 *G. max* (cultivated soybean)	×5 depth	SOAP	6,318,109	Lam et al., 2010
3	25	8 *G. soja*, 17 *G. max* (8 landraces, and 9 elite lines/cultivars)	–	SOAP	5,102,244	Li et al., 2013
4	16	10 *G. max* and 6 *G. soja*	>14x	GATK	3,871,469	Chung et al., 2014
5	7	*G. soja*	~111.9X	SOAP	3.62–4.72 M SNP per line	Li et al., 2014
6	11	10 Semi-wild and 1 *G. soja*	9 Semi-wild at ~3X while 1 Semi-wild at ~41X, and 1 Wild at ~55X	SOAP	7,704,637	Qiu et al., 2014
7	302	62 *G. soja*, 240 *G. max* (130 landraces, and 110 improved cultivars)	>11X	GATK	9,790,744	Zhou et al., 2015

**Figure 2:**
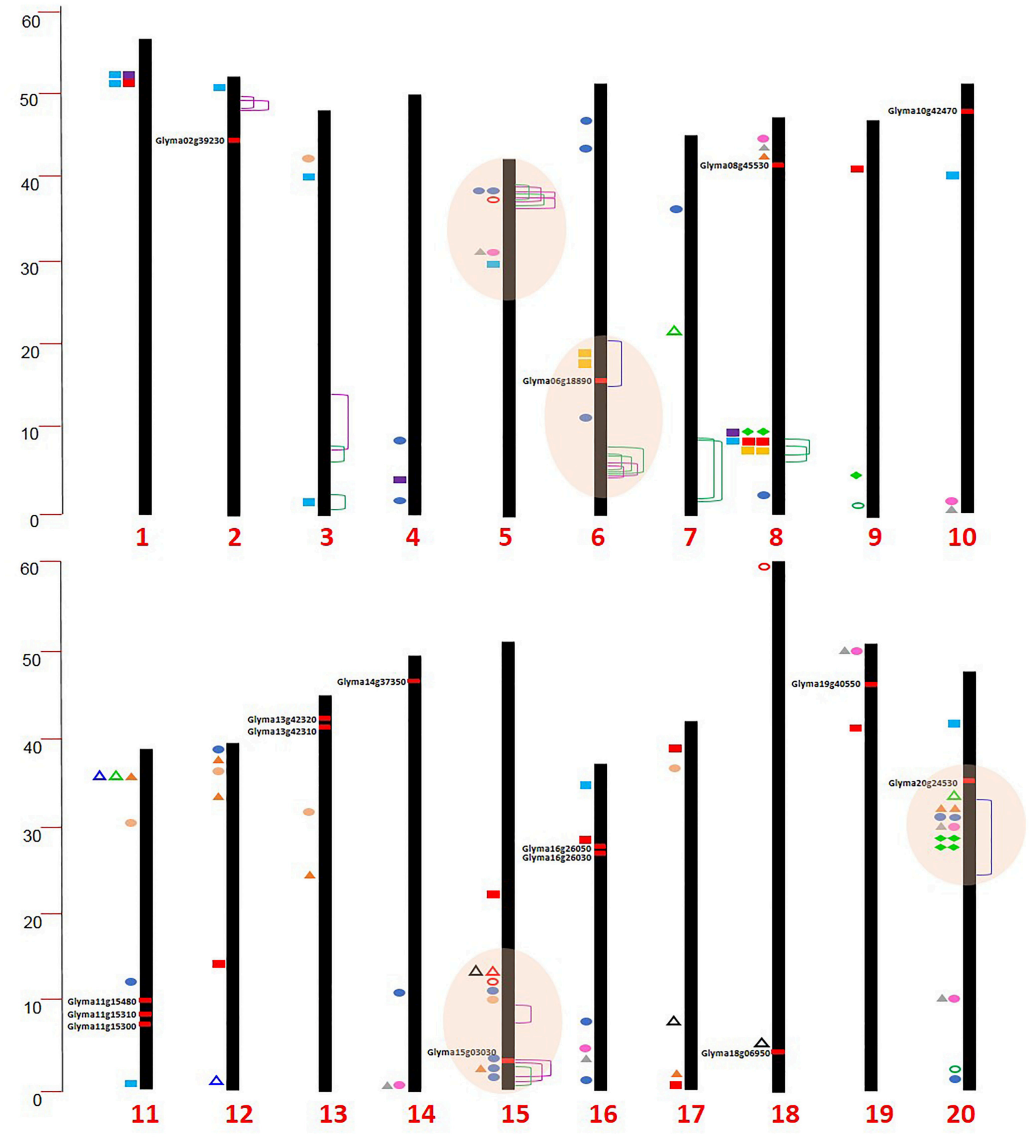
**Chromosomal locations of genomic hot-spots, promising genes, QTL, GWAS, and linked markers for soybean seed composition from several studies**. 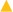
Protein, 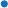 Oil, 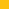 Cysteine, 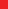 Lysine, 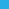 Methionine, 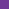 Threonine, 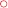 Sucrose, 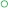 Stachyose (Vaughn et al., 2014); 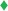 Protein and oil (Hwang et al., 2014); 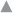 Protein, 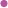 Oil (Sonah et al., 2015); 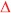 Glucose, 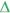 Sucrose, 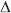 Fructose, 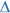 Stachyose (Wang et al., 2014b); 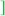 Protein, 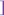 Oil (Pathan et al., 2013), 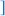 Protein, Methionine (Warrington et al., 2015).

Researchers have shown that fatty acid composition and protein content of soybean seed are largely affected by environmental factors (more specifically temperature), which challenges the efforts in phenotyping (Bellaloui et al., 2015). To overcome this problem, MAS for fatty acid has been successfully employed in several breeding programs (Pham et al., 2010, 2012, 2014). Precise mapping of QTL by high-throughput genotyping platforms has revolutionized MAS for soybean seed trait improvement (Supplementary Tables 1, 2; Supplementary Figure 2). In a recent study, Warrington et al. (2015) identified a major QTL on Chr. 20 (55% phenotypic variation) for seed protein and amino acid content in a Benning × Danbaekkong population; however, a negative correlation between total protein and amino acid (especially for Thr) was observed. Information about genomic loci governing seed composition traits collected from QTL, genome-wide association study (GWAS) and WGRS studies from the past few years is summarized to locate genomic hot-spots in chromosome locations. Interestingly, a majority of identified QTL for seed composition traits were found to be on Chrs. 20, 15, 6, and 5 (Supplementary Table 1; Figure 2). The QTL on Chr. 20 explained 12–55% of phenotypic variation associated with increased protein content. Compilation of data showed that several genomic loci for oil and protein from different studies were found to be co-localized. This confounding region could provide an entry point to investigate the basis of correlation between various seed composition components and could be useful for gene pyramiding strategies.

Several efforts of meta-QTL analysis have been performed for seed composition traits in soybean (Zhaoming et al., 2009; Sun et al., 2012). QTL meta-analysis combines datasets from independent studies to detect consensus QTL and to shrink the QTL confidence intervals making them more useful for MAS (Rudner et al., 2002). In soybean, there are several meta-QTL studies that have been conducted for seed traits; for example, 17 meta-QTL have been identified for hundred-seed weight (HSW) using 65 QTL from 12 studies (Zhaoming et al., 2009). In another study, targeting HSW by multi-environmental mapping followed by meta-analysis, 15 consensus QTL were identified (Sun et al., 2012). In addition, seed oil content was also examined through meta-QTL analysis (Qi et al., 2011a,b). Similarly, Zhao-Ming et al. (2011) performed meta-QTL analysis for seed protein content and reported 23 consensus QTL by integrating 107 QTL. In summary, these studies identified hotspots for seed traits and this could be helpful to improve seed composition traits. Meta-QTL analysis gives the basis for gene mining and also facilitates refining soybean genetic maps. However, meta-QTL may indicate presence of pleotropic traits by creating QTL groups or clusters for several traits which necessitates confirmation of hotspots by integration with other approaches.

### Association mapping

QTL mapping is routinely conducted and requires mapping populations derived from bi-parental crosses, such as F_2_, backcross (BC), or recombinant inbred lines (RILs). Despite the success of QTL mapping, it is limited by a narrow range of allelic diversity and roughly estimated QTL intervals, which refers to large genomic regions (Borevitz and Nordborg, 2003). Therefore, to address this issue, a statistically more advanced method, GWAS has been developed. GWAS utilizes allelic diversity in large sets of germplasm and the recombination events accumulated over hundreds of years during the evolution and domestication. A few merits of the GWAS approach includes high resolution, often to the gene level, and the use of previously well studied populations that provide a correlation between genetic and phenotypic variations. In addition, GWAS provides statistical power to reconnect the phenotype to its underlying genetics (Brachi et al., 2011). A number of GWAS have been performed for various traits in soybean with great success in identifying loci with high mapping precision (Supplementary Table 3). Vaughn et al. (2014) used GWAS to identify genetic loci with < 1 Mbp resolution governing the soybean seed composition traits. In this study, the seed oil, protein, amino acids, sucrose, and oligosaccharide (RFO's) content were dissected to identify the precise position of genes. A study conducted to explore the genome wide association between seed protein and oil content in soybean revealed 40 SNPs associated with protein and 25 SNPs associated with oil content (Hwang et al., 2014). In addition, Zhou et al. (2015) used high density SNP data with WGRS and identified more precise regions for seed oil content, seed weight, seed coat color, and domestication traits. GWAS can be used in place of QTL mapping to eliminate the limitation of bi-parental mapping populations. However, GWAS has its own merits and demerits (Korte and Farlow, 2013). Combining GWAS with QTL mapping will provide advantages for precise mapping and understanding of genetic architecture of seed traits (Figure 2). Recently, Sonah et al. (2015) demonstrated the integrated approach of QTL mapping and GWAS for the identification of loci/candidate genes for various traits including seed weight, oil, and protein content in soybean. A total of 25 loci were identified, including three loci associated with seed weight and eight each for oil and protein content.

Despite GWAS's merits, it has some limitations in the detection of rare alleles that are associated with natural genetic variation, which may lead to obscure analysis and synthetic associations (Dickson et al., 2010; Platt et al., 2010). Even though a considerable amount of false positives in GWAS can be reduced by using a correction of population and kinship approach, such errors cannot be fully eliminated. This is particularly true when the subpopulations are extensively diverse. However, association studies are genuinely promising to identify significant genotype-phenotype correlations comprehensively for complex traits (Hwang et al., 2014; Vaughn et al., 2014). It is foreseeable that the initial success in candidate gene identification using an association approach would greatly help in the advancement of the deeper understanding of complex soybean seed traits.

### Genomic selection

Association studies and MAS have been used in plant improvement programs for several years. Nevertheless, they have some limitations, such as long selection cycles and the identification of significant marker-QTL associations which is unable to capture “minor” gene effects (Desta and Ortiz, 2014). These limitations can be effectively addressed by using a promising approach, known as genomic selection (GS) (Meuwissen et al., 2001). Unlike MAS, GS utilizes the entire set of markers to predict genomic estimated breeding values (GEBVs) for all genotyped individuals within a breeding population by capturing the total additive genetic variance for a particular trait of interest (Heffner et al., 2009, 2011). GS requires a training population (both genotyped and phenotyped) to calculate breeding values by using all marker information and avoiding biased marker effects simultaneously (Heffner et al., 2009). The primary benefit of GS is that selection can be imposed at a very early stage in the breeding process, thus accelerating the breeding cycles without phenotyping. However, optimizing the constituents of a training population is very challenging and is influenced by a number of parameters, such as choice of model, size of training data, trait heritability, span of linkage disequilibrium (LD), marker density, and strength of genetic relationships between training and validation populations (Bentley et al., 2014).

Due to the advantages of GS over conventional methods, it has been successfully applied to a variety of crops, such as wheat and maize (Crossa et al., 2010), pear (Iwata et al., 2013), sugar beet (Würschum and Kraft, 2014), and is constantly being used for other crop species. There are only a few reports for the application of GS in soybean (Hu et al., 2011; Shu et al., 2012; Bao et al., 2014; Jarquín et al., 2014). Hu et al. (2011) studied primary embryogenesis capacity in soybean using 126 RILs and 80 SSRs and reported a strong correlation (*r*^2^ = 0.78) between GEBVs and phenotypic data (Hu et al., 2011). A study was performed on soybean HSW using 79 sequence-characterized amplified region (SCAR) markers for 288 varieties. This study reported a correlation coefficient of 0.904 amongst the GEBVs and phenotypic values (Shu et al., 2012). Recently, Bao et al. (2014) investigated soybean cyst nematode resistance via genotyping of 282 accessions employing the 1536 SNP array and demonstrated a significant prediction accuracy (0.59–0.67) for soybean cyst nematode resistance in soybean. Another study reported a prediction accuracy of 0.64 for grain yield, which indicates a good potential of GS utilization strategy in soybean (Jarquín et al., 2014). It is noticeable that implementation of a GS approach is needed in soybean seed related traits. Moreover, with the declining genotyping costs and increasing phenotyping costs, GS can be helpful to mitigate many of the selections associated with phenotyping and ultimately speed up the breeding cycles. Hence, the GS statistical method is more feasible and more emphasis should be given to employ this approach in the improvement of seed composition with available genotypic and phenotypic data.

## Soybean seed transcriptomics advancements

Regulation of gene expression occupies a central role in the flow of genetic information. Location and level of gene expression gives the insight in the functional regulation, thus, by collecting and comparing transcriptome of different tissue-types, stages or development, researchers can gain a deeper understanding of how changes in transcription may affect the phenotype. There are several tools for generating and mining the transcriptomes including gene-by-gene and global methods for quantification of expression levels (Wirta, 2006). Global methods allow for a nearly comprehensive analysis of the transcriptome, which comprise of hybridization-based (microarrays and GeneChips); sequence tag-based [ESTs sequencing, cDNA deep sequencing, serial analysis of gene expression (SAGE), and massive parallel signature sequencing (MPSS)], and RNA-sequencing (RNA-seq) approaches. Chip-based technologies involving microarrays have become a dominant platform after ESTs sequencing and genome sequencing in several plant species.

The RNA-seq approach provides information of large-scale sequences of coding and non-coding RNAs without prior genomic information. RNA-seq data captures transcriptome dynamics across different tissues without data set normalization and has been applied in the identification of genes and regulatory networks associated with soybean seed composition (Severin et al., 2010; Goettel et al., 2014). It also has considerable advantages in examining novel transcript, splicing events, and allele-specific expression. Due to these advantages, RNA-Seq is considered a valuable technology in understanding transcriptomic dynamics during developmental and physiological changes (Severin et al., 2010).

To date, several studies have been performed to track gene expression changes from fertilization to maturity in soybean seed development events utilizing RNA-Seq (Severin et al., 2010). For instance, a genome-wide expression analysis was performed for soybean MADS gene family using publicly available RNA-seq data from 17 tissues (Fan et al., 2013). Similarly, the public RNA-seq database was utilized to study sugar transporter genes (SWEET) in soybean seed and other tissues (Patil et al., 2015). This study identified several SWEET genes, which are highly expressed, and plays an important role in nutrient unloading during seed development and seed filling stages. Significant efforts have been made in understanding gene function and regulation using transcriptome profiling (Table 2). Recently, O'Rourke et al. (2014) used RNA-seq to analyze oil biosynthesis, nitrogen assimilation, and transcription factors affecting oil, carbohydrate, and protein deposition during seed fill. In another study, Goettel et al. (2014) investigated the transcript polymorphism in soybean lines varying in oil composition and content. They demonstrated a high correlation of transcript and genetic variation associated with oil quality traits. In addition, Yin et al. (2014) compared soybean seed specific genes from microarray, DDD (differential digital display), and RNA-seq databases and identified 184 seed development specific genes. Most of the identified genes were found to be related to nutrient reservoir activity, lipid binding, enzyme inhibitor activity, peptidase regulator activity, hydrolase activity, embryo development, lipid transport, proteolysis, vacuoles, or lipid particles. Due to the large number of gene expression studies performed on soybean seed, a huge amount of data is available in the Gene Expression Omnibus (GEO) at National Center for Biotechnology Information (NCBI) database (Supplementary Figure 3). The large amount of microarray and deep sequencing transcriptomic data have allowed the development of a soybean co-expression network database containing 23,267 genes, 1873 miRNA-target pairs, and a group of acyl-lipid pathways containing 221 enzymes and more than 1550 genes (Yu et al., 2014).

**Table 2:** **List of significant seed related gene expression studies in soybean**.

**Purpose of the study**	**Tissue stages/condition**	**Approach**	**References**
Gene expression during embryo development and isoflavonoid biosynthesis	30, 40, 50, 60, and 70 DAP	Microarray	Dhaubhadel et al., 2007
Expression profile of cotyledon during seedling development	Imbibed underground seed for 48 h; radicle 10–15, 16–25 mm long; hypocotyl 40–50 mm; green and yellow cotyledon above the ground, cotyledon mostly green, cotyledon fully green	Microarray	Gonzalez and Vodkin, 2007
Identification of differentially expressed genes in soybean seeds differing in oil content	15, 22, 29, 36, and 43 DAF	Microarray	Wei et al., 2008
Seed developmental stages from mid-maturation to full maturation	25–50, 75–100, 100–200, 400–500 mg at green stage; 200–300 mg at yellow; 100–200 mg at dry seed	Microarray	Jones et al., 2010
High quality gene expression in 14 diverse tissues (aerial, underground and seed tissues)	Pod 7, 10–13, 14–17 DAF; Seed 21, 25, 28, 35 DAF, Roots after 12 DAI; Nodules at 20–25 DAI	RNA-seq	Severin et al., 2010
Seed developmental stages and gene expression profiles	10–50 DAF (with 5 day interval)	Microarray	Sha et al., 2012a
Explore genes involved with lipid biosynthesis during seed development	15 DAF, and then every 5 days until 70 DAF	RNA-seq	Chen et al., 2012
Change in gene expression pattern from the beginning of seed formation	Pod 1 WAF, bean 2-mm and 5-mm, and 12–14 mm	Microarray	Asakura et al., 2012
Tissue and seed developmental stage specific MicroRNAs (miRNAs) identification	25–50 mg, 100–200 mg green; 300–400 mg yellow seed	MiRNA sequencing (Degradome Sequencing	Shamimuzzaman and Vodkin, 2012
Seed development from fertilization to maturity	4, 12–14, 22–24 DAF whole seed; 5–6 mg whole seed; 100–200, 400–500 mg cotyledon, 100–200 mg whole seed, dry	RNA-seq	Jones and Vodkin, 2013
Seed transcriptomes from nine soybean genotypes varying in oil composition and content, and to identify sequence variation in seeds at gene, pathway and systems levels	S6 stage of seed	RNA-seq	Goettel et al., 2014
Gene expression patterns for seed protein and oil synthesis during seed development	1 and 2 WAF	RNA-seq	Jang et al., 2015

*DAF, Days After Flowering; WAF, Week After Flowering; DAI, Days After Inoculation; DAP, Days After Pollination*.

The wild soybean (*G. soja*) is the unique resource to study the regulation of seed composition traits (specifically oil and protein) because *G. max* produces nearly twice as much oil and less protein than *G. soja*. The difference in seed oil content and composition within soybean germplasm is largely affected by genomic variation and expression profile of the genes involved in fatty acid biosynthesis and other unknown regulators. Until now, several wild soybean accessions have been re-sequenced (Kim et al., 2010; Joshi et al., 2013; Li et al., 2014); however, they have not been studied at the transcriptome level, specifically during the seed developmental stages. Sequence variants and expression polymorphism associated with gene function can help in dissecting the underlying causes of phenotypic variation.

## Proteomics advances

Proteomics is the large-scale study of structural and functional features of a set of proteins present in an organism. Proteomics has gained ample popularity over the genome-based technologies as it directly deals with biochemical processes. Moreover, post-translational modifications of protein can also be studied through proteomic techniques (Eldakak et al., 2013). Soybean seed proteome is dominated by two major storage proteins, glycinin (11S legumin type) and conglycinin (7S vicilin type), and it also includes many moderately abundant proteins, such as the kunitz trypsin inhibitors, lectin, P34 allergen, sucrose binding protein, urease, oleosins, and several thousand low abundance proteins (Herman, 2014). Due to the complexity of soybean seed proteins, several proteomic studies have been conducted to understand the protein expression, functions, and interactions. Gel-based, mass-spectrometry based methods or a combination of techniques have been tested in soybean to study global changes during seed development (Nouri and Komatsu, 2010; Oh et al., 2014). However, gel-based methods, such as, two-dimensional gel electrophoresis (2-DGE) is considered a time-consuming technique with poor sensitivity and reproducibility as compared to gel-free methods. Recently, a combinatorial approach, NanoUPLC-MS^E^ (liquid chromatography combined with mass spectrometry) procedure was used for the assessment of peptide profile in soybean seeds and compared with the Uniprot database (Murad and Rech, 2012). In another study, the high yielding cultivar, Jidou17 and its parental lines were used to examine differentially expressed proteins using an iTRAQ-based (isobaric tags for relative and absolute quantitation) method (Qin et al., 2013). All of these methods are considered to be high-throughput, accurate, sensitive, and more robust than gel-based techniques.

Soybean seed traits have been exploited using proteome profiling with gel-based and gel-free techniques, e.g., peptide mass fingerprinting of seed proteins was performed using 2-DGE and resulted in detection of approximately 150 seed proteins. Most of them were found to be related to seed storage proteins (Mooney and Thelen, 2004). A seed filling protein profile was analyzed at 2, 3, 4, 5, and 6 weeks after flowering using 2-DGE and MALDI-TOF (matrix-assisted laser desorption ionization time-of-flight) mass spectrometry. This study identified 422 proteins including 216 non-redundant proteins. Most abundant proteins were found to be involved in metabolism, protein destination and storage, metabolite transport, and disease/defense (Hajduch et al., 2005). Agrawal et al. (2008) studied the protein profile associated with seed filling using 2-DGE and semi-continuous multi-dimensional protein identification technology (Sec-MudPIT) coupled with liquid chromatography-mass spectrometry. A total of 478 non-redundant proteins were identified, which were mainly involved in metabolism, protein destination and storage (Agrawal et al., 2008). In another study, Barbosa et al. (2012) compared the expression patterns of seed proteins of transgenic and non-transgenic soybean using 2-D DIGE (difference gel electrophoresis), MALDI quadrupole time-of-flight (QTOF) and electrospray ionization (ESI) QTOF. The phosphoproteomic profile of soybean, rapeseed, and Arabidopsis seed at five developmental stages was analyzed. A total of 2001 phosphopeptides containing 1026 unambiguous phosphorylation sites from 956 proteins were identified. In comparison with other large-scale phosphoproteomic studies, 652 of the phosphoproteins were found to be novel. The unique proteins fall into several gene ontology categories, some of which were found to be involved with metabolic processes, RNA binding, and embryonic development (Meyer et al., 2012). Stored nutrients and their mobilization in soybean seed determines the early plant vigor and these reserve components can be determined by proteomic profiling. A proteome based study revealed different mechanisms for reserve mobilization in soybean and rice during germination (Han et al., 2013).

Technological advances in proteomics methods offer more sensitivity, greater rapidity, and proteome coverage. These techniques and generated data can potentially help to acquire a comprehensive understanding of the physiology of seed reserves. However, the data processing and analysis are still a bottleneck in proteomics studies. To overcome this, it is important to integrate publicly available data and bioinformatics tools into a more robust linear pipeline for soybean seed trait improvement.

## Metabolomics advances

Metabolomic studies provide a high-throughput assessment of all metabolites, which represent the complete set of small molecules in the target tissue or organism. Small molecule metabolites in plant composition can be analyzed using metabolomics in combination with sophisticated statistical and computational methods. A metabolomic approach has been widely used to dissect biochemical composition and regulation in plant seeds, such as in rice (Matsuda et al., 2012), maize (Rao et al., 2014), and tomato (Toubiana et al., 2015). Soybean seed contains flavonoids, isoflavones, saponins, phytosterols, and several other metabolites that have a considerable impact on human health. Metabolomics is an emerging field, which has been used to assist in the biochemical analysis of complex mixtures and considered a robust, sensitive, and powerful technology (Nakabayashi and Saito, 2013). Fukusaki and Kobayashi (2005) explained the technical elements, statistical analysis, and practical applications while Putri et al. (2013) elaborated on the latest developments in analytical methods and data analysis in the metabolomics area.

García-Villalba et al. (2008) developed a metabolic profiling method for genetically modified (GM) and conventional soybean lines. This method identified more than 40 compounds and interestingly showed significant quantitative differences between conventional and GM soybean lines. In another study, the soybean seed metabolome (169 metabolites) was assessed in GM and conventional lines. Interestingly, no significant variation was observed between the seed metabolomes except in the engineered targeted pathway (Clarke et al., 2013). In addition, metabolite-metabolite interaction was studied in seed; and a seed metabolic network map was constructed based on 169 metabolites from 29 soybean cultivars. This might be helpful for metabolic engineering to enhance seed quality in soybean (Lin et al., 2014).

Advances in database development and bioinformatics tools are still lagging behind for seed metabolomics area. A database model, ArMet (an architecture for metabolomics) was designed for Arabidopsis and *Solanum tuberosum* metabolomics studies (Jenkins et al., 2004). SoyMetDB (http://soymetdb.org) has been developed for integrating, mining and visualizing metabolomic data from soybeans (Joshi et al., 2010). The potential findings of various metabolic studies performed on different soybean tissues will provide a basis to improve soybean seed quality and perhaps yield. To date, studies focusing on seed specific metabolites are limited; therefore, there is a dire need to create a comprehensive metabolome of the soybean seed.

## Phenomics development

Plant phenotyping is a process of recording quantitative and qualitative plant traits and has been the backbone of breeding programs to improve desired traits. Phenotype includes a set of morphological, structural, physiological, and biochemical traits that characterize a genotype at a given stage, date, or environment. High-throughput genotyping technologies have provided a quantum of genomic information for individual lines and large mapping populations. However, the phenotypic information is not as extensive and provides limited contribution to the advancement of crops due to the labor intensive, time-consuming task of collecting phenotypic data. Plant breeding strategies call for high-throughput, rapid, and accurate phenotyping developments particularly due to the utilization of more crosses, replications, and environments (Araus and Cairns, 2014). Genotypic information is a selection criteria in advanced statistical models for trait improvement but phenotypic data is required at the initial selection of lines which necessitates the development of phenotypic data collection methods. The genomic prediction model is one such example that uses genotypic information for selections; however, the phenotypic information is needed to train a prediction model (Lorenz et al., 2011). The advancements of high-throughput measurement methods of plant traits have helped breeding in various ways. For example, integration of high-throughput phenotyping (HTP) with association studies will be helpful in trait discovery and phenotypic predictions.

HTP platforms have seen advances in non-destructive and time-series based methods. Some examples include image-based computer vision phenotyping, image processing, data extraction tools, and the availability of public datasets. The techniques available due to recent advances provide platforms for data measurement during different growth stages of individual plants or large mapping populations. Attempts to exploit phenotypic measurements, new software and hardware tools are available such as BreedVision (Busemeyer et al., 2013), TraitCapture (Brown et al., 2014), and Pheno-Copter (Chapman et al., 2014). The major seed composition traits in soybean including total oil, protein, fatty acids, carbohydrate, ash, and moisture can be measured using high resolution nuclear magnetic resonance (HR-NMR) and near infrared (NIR) methods (Baianu et al., 2012). A soybean seed composition database for approximately 15,000 accessions is available with protein, amino acid, oil, fatty acid, isoflavone, carbohydrate, fiber, and moisture data. This data was collected from over 80,000 spectroscopic NIR and NMR measurements from both bulk and single soybean seed samples (Baianu, 2011).

Baianu et al. (2012) first reported the HR-NMR for the determination of amino acids from whole soybean seeds without seed protein extraction and they also reported the calibration models, methodologies, and validation procedures for the measurement. The latest NIR grain analyzer (FOSS) is evolving with the modern electronics and precision optical components for quality data. In a recent study, crude protein and amino acid were determined by utilizing NIR to identify QTL for these two traits (Warrington et al., 2015). Other than seed oil and protein composition, the other components, such as lipoxygenases and secondary metabolites can be measured using low throughput methods. Seed lipoxygenase produces an unpleasant beany flavor and this can be measured with colorimetric assays and single-dimension sodium dodecyl sulfate polyacrylamide gel electrophoresis (SDS-PAGE) (Suda et al., 1995).

Besides the seed composition traits, another study used a laser light back-scattering imaging technology to identify specific soybean cultivars through charge-coupled device (CCD) camera images (Zhu et al., 2012). In this approach, soybean seed surface was directly illuminated by laser light, and later using image processing technology to categorize different cultivar seeds. Progress in phenotypic characterization falls far behind when compared to the progress made in the high-throughput and automated genotyping techniques. Therefore, there is a need for advancing phenotyping tools and databases development to eliminate the setbacks in the research programs aiming to develop better quality soybeans.

## Ionomics improvement

The advances in the areas of genomics, proteomics, metabolomics, and phenomics have led to the development of elemental profile analysis as well, known as “ionomics.” Ionomics can be used for the analysis of physiological, biochemical, elemental, and mineral profiling in living systems with a high-throughput and cost effective method (Baxter, 2010). The elemental composition of soybean seed is an important component of their overall nutritional value and is controlled by element availability during seed development. Hence, understanding the ionome of soybean seed and its correlation with genetic factors has the potential to improve seed composition and nutrient values. Typically, the conventional methodologies for elemental analysis are based on either the electronic properties of an atom (emission, absorption, and fluorescence spectroscopy) or nuclear properties (radioactivity or atomic number; Salt et al., 2008). In addition, advances in mass spectroscopy technology, such as inductively coupled plasma (ICP), has helped in multiple element analysis and enables complete ionome instead of just individual elements.

Ionomics has been used to compare transgenic and non-transgenic soybeans (Yan et al., 2007; Mataveli et al., 2010, 2012). Similarly, another study proposed a combination of microwave-induced combustion (MIC) and ICP-MS to analyze bromine, chlorine, iodine, and the associated products in soybeans (Barbosa et al., 2013). Recently, a high-throughput approach was utilized to identify seed elemental composition in 947 mutagenized lines (Ziegler et al., 2013). In this study, they identified mutants with modified seed element profiles. Sha et al. (2012b), performed ionome analysis of soybean seed and concluded that the seed ionome is affected by the cropping system and manure applications. In addition to the essential elements, many crop plants including soybean, may accumulate the non-essential or toxic elements such as cadmium and lead (Sugiyama et al., 2011). With the advantages of low cost, high-throughput capabilities compared to the proteomics and metabolomics, ionomics became a powerful approach to understand complex biological systems controlling elemental accumulation in plants. Thus, ionome study can be utilized as an effective tool to build connections not only with genome, metabolome, and physiological processes of plant, but also with environment and ecology for elemental variation between genotypes, loci/genes identification.

## Genetic engineering

The availability of the sequence information provides new avenues for the engineering of soybean seed composition. The new “omics” approaches offers a potential resource to comprehend the metabolic regulatory network governing seed storage compound accumulation. To accommodate the global food demand and growing population needs, several genetic engineering and genome editing strategies need to be employed to obtain the next-generation crop at faster rate than the conventional breeding. Studies have reported improved soybean seed oil content via X-ray, and EMS-induced mutations in the desired genes (Dierking and Bilyeu, 2009; Pham et al., 2010, 2011). For example, deletion of a 100 kb sequence on Chr. 10 in a mutant line M23 increased oleic acid content but caused yield drag (Pham et al., 2011). Similarly, the SACPD-C deletion in an A6 mutant line showed elevated stearic acid content; however, simultaneously a reduced seed yield was also observed (Gillman et al., 2014). Nevertheless, conventional methods require labor intensive screening procedure to identify germplasm accessions with desired phenotypes and stable performance in multiple generations and environments.

Genetic engineering entails a range of activities for the benefit of agriculture, such as creation of genetically modified (GM) or transgenic crops. A number of transgenic approaches have been explored for modifying the seed composition traits, including total oil (Lardizabal et al., 2008), protein (Schmidt et al., 2011), oleic acid (Haun et al., 2014), phytosterols (Neelakandan et al., 2012; Nguyen et al., 2013), tocopherols (Karunanandaa et al., 2005), and isoflavone (Yu et al., 2003). Marginal enhancement of total oil content was achieved by seed-specific expression of DGAT2 enzyme from oil accumulating fungi (Lardizabal et al., 2008). In another study, isoflavone levels in soybean seeds were increased via metabolic engineering of the complex phenylpropanoid biosynthesis pathway (Yu et al., 2003). Various techniques, such as transgene-mediated RNA interference (RNAi), has been successfully employed by researchers in enhancing soybean seed oil (Buhr et al., 2002; Wagner et al., 2011).

The genetically modified organisms (GMOs) have quite a few limitations, such as social acceptance, cost-related concerns (regulatory and licensing), limited resources, and time. To overcome these drawbacks, genome editing has arisen as a modern approach for specifically targeting and modifying DNA sequences for crop improvement and is considered a non-GM approach (Voytas and Gao, 2014). Several methodologies are available for genome engineering, such as engineered homing endonucleases or meganucleases, zinc finger nucleases (ZFNs) and transcription activator-like effector nucleases (TALENs). These are being used to mutagenize genomes at precise locations (Cermak et al., 2011; Sander et al., 2011). An optimized mutagenesis approach is required for the simultaneous recovery of plants with single or multiple mutations in a gene of interest without interfering with the rest of the genetic background. Therefore, site-directed mutagenesis could be a powerful method for producing the desired results. Moreover, CRISPR (clustered regularly interspaced short palindromic repeats)/Cas (CRISPR-associated) has emerged as a simpler method that permits introduction of desired SNP into a gene of interest without a need for extensive design and time-consuming assembly of individual DNA-binding proteins (Belhaj et al., 2013; Bortesi and Fischer, 2014; Voytas and Gao, 2014). In addition, Cas9 has been reported as a successful method for genome editing in tobacco (Nekrasov et al., 2013), rice, wheat, Arabidopsis, tobacco, and sorghum (Jiang et al., 2013), and sweet orange (Jia and Wang, 2014). Targeted genome editing has been tested for soybean seed composition improvement. The context-dependent assembly (CoDA) was used to create ZFNs for target specific mutagenesis (*DICER-LIKE* and other genes involved in RNA silencing) in soybean. This approach showed successful heritable transmission of the ZFN-induced mutation in subsequent generations and could be useful for making mutations in duplicated genes efficiently (Curtin et al., 2011). While in another study, mutations were induced in two fatty acid desaturase-2 genes (*FAD2-1A* and *FAD2-1B*) using TALENs. The changed fatty acid profile of the seed with increased oleic acid (20–80%) and decreased linoleic acid (from 50% to less than 4%) was reported for *FAD2-1A* and *FAD2-1B* mutations (Haun et al., 2014). In comparison to other transgenic technologies, the CRISPR system is considered a non-GM technology, since the CAS9 can be deleted from the host plant via backcrossing in subsequent generations, once the mutation is accomplished. The usefulness of the CRISPR system being a simple and cost effective technique for genome editing in soybean can't be denied. This approach can be applied to confirm candidate genes, novel alleles/phenotypes, and engineer soybeans with high quality seed traits.

## Integrated “omics” approaches

With the improvements in different “omics” approaches and the development of computational tools; this has provided information related to gene function, genome structures, biological pathways, metabolic and regulatory networks and has greatly contributed to the understanding of plant systems. Although several biological network models are available, they do not always provide a complete depiction of cellular and molecular networks solely based on genome, transcriptome, or metabolome data. In the soybean research community, several groups have re-sequenced soybean germplasm (Chung et al., 2014; Li et al., 2014; Qiu et al., 2014; Zhou et al., 2015). Their studies were focused on population genetics and also to understand the domestication process of cultivated soybeans (Table 1). Secondly, integrated approaches have not been explored to study the interacting networks between genomic sequences and transcript profiles. Thus, an integrated data analysis approach is essential to investigate and fully understand the physiological, biochemical, and molecular interactions. For instance, QTL mapping and GWAS are helpful tools in the identification of chromosomal regions that relate to phenotypic traits; however, underlying genes in that region are in large numbers and the candidate gene is not identified with QTL or GWAS alone (Deshmukh et al., 2014). The integrated genomic approach, combined with WGRS data and seed development related transcriptome for diverse germplasm and RILs could identify a novel/uncharacterized variant-linked co-expression network associated with seed composition traits.

In soybeans, a combination of these approaches has led to successful discoveries, for instance, Kovinich et al. (2011) combined gene expression and metabolite data to elucidate the control of the R locus identification of pigment biosynthesis genes (Kovinich et al., 2011). In a similar study, metabolic and transcriptional changes were assessed in developing soybean seeds to identify metabolic engineering targets. The study concluded that transcriptional activation and signaling involvement was much higher during seed maturation and dormancy (Collakova et al., 2013). The integration of expression QTL (eQTL) and phenotypic QTL (pQTL) was employed in the identification of genes for isoflavone content in soybean seeds and 11 potential candidate genes were identified (Wang et al., 2014a). Recently, ionomics and metabolomics were coupled for a comprehensive assessment of GM and non-GM soybean lines (Kusano et al., 2015). Li et al. (2015) found the highest metabolic flux during early seed fill by integrating metabolomics and transcriptomics analysis. Furthermore, the metabolic flux was found to be consistent with regard to the transcript and metabolite level changes during the seed development stages. All of these studies clearly illustrated that an integrated “omics” approach needs to be applied for better understanding of seed composition traits in soybean.

## Available online resources

Recent advancements in the “omics” methodologies and approaches in the past decade has provided a large amount of data with the objective of discovering key genes associated with phenotypic traits. The acquired data and information are available at several public databases, which facilitates sharing of the generated information. The availability of integrated and focused databases from different dimensions have allowed phenotypic trait improvement in soybean as well. The online available databases include user-friendly interfaces, such as chromosomal visualizer, omics datasets, computational comparisons, and search tools that allow easy data analysis for a particular objective. A considerable number of soybean information resources are freely accessible and are summarized in Table 3. The available data in the public databases for various applications will facilitate and accelerate molecular elucidation of cellular system linked to agronomically important traits.

**Table 3:** **List of wide-ranging available online resources for soybean**.

**Web Name**	**URL**	**Description/Tools/Applications**	**References**
Soybean Genomics and Microarray Database (SGMD)	http://bioinformatics.towson.edu/	Genomic, EST, and microarray database	Alkharouf and Matthews, 2004
Soybean transcriptome database (SoyXpress)	http://soyxpress.agrenv.mcgill.ca	Metabolic pathways, EST sequences, Microarray and Affymetrix gene expression data	Cheng and Strömvik, 2008
Soybean Proteome Database	http://proteome.dc.affrc.go.jp/Soybean/	Proteome, Metabolome, Transcriptome datasets, 2D-PAGE and proteomics information, comparative proteomics under flooding, drought and salt stress.	Sakata et al., 2009
SoyBase	http://www.soybase.org	Genetic and physical maps, Genome sequence, Transposable elements, Annotations, Graphical chromosome visualizer	Grant et al., 2010
Soybean Knowledge Base (SoyKB)	http://soykb.org/	Graphical chromosome visualizer, Genes/proteins, miRNAs/sRNAs, Metabolite profiling, Molecular markers, Plant Introduction and traits information	Joshi et al., 2012
Soybean proteins database (SoyProDB)	http://bioinformatics.towson.edu/Soybean_Seed_Proteins_2D_Gel_DB/Home.aspx	Seed protein identification, 2D gel image data	Tavakolan et al., 2013
Soybean Cyst Nematode proteins database (SCNProDB)	http://bioinformatics.towson.edu/	SCN protein identification, 2D gel images data	Natarajan et al., 2014
Soybean Functional Network (SoyFN)	http://nclab.hit.edu.cn/SoyFN	Functional gene network, microRNA functional network, gene annotation, genome browser	Xu et al., 2014
Soybean Functional Genomics Database (SFGD)	http://bioinformatics.cau.edu.cn/SFGD/	Gbrowse, microarray expression profiling, transcriptome data, gene co-expression regulatory network, acyl-lipid metabolism pathways, cis-element significance analysis	Yu et al., 2014

## Concluding remarks

The advancements in NGS technologies have made huge amounts of sequence information publicly available and now it is time to integrate and utilize them for the improvement of seed composition and other traits (Table 1; Figures 1, 2). Hundreds of QTL for seed oil, protein, and other seed component traits have been identified and reported; however, very few studies were incorporated in breeding programs (Supplementary Table 2). The negative correlation, marginal improvement, and low stability across different geographical locations have undermined the development of cultivars with high meal quality, oil and protein content, and yield. However, for protein improvement with better yield and maintained oil content, attempts are being made by utilizing identified QTL. Most of the commercial soybean cultivars are fixed for the low protein allele at the major QTL on Chr. 20, suggesting that introgression of desired allele at Chr. 20 into an existing elite background would enhance protein content. Also, since there is no single commercial soybean cultivar with the FAO standard for total sulfur containing amino acids; therefore, it presents an opportunity for soybean researchers to improve nutrient values of soybean seed using integrated approaches. It is essential that breeding efforts should be made considering the overall improvement of protein, oil, and yield traits in soybeans. As evident from the discussion above, advances in “omics” need to be integrated, particularly in genomics, proteomics, metabolomics, phenomics, and ionomics for soybean seed composition. Strategies to dissect the inverse relationship and environmental stability of seed storage protein with oil and yield needs to be designed. This could be achieved by uniting “omics” and conventional approaches.

A number of potential strategies have been outlined, including combining diverse and wild germplasm associated with specific seed traits and studying them in various “omics” dimensions to enhance our knowledge. It is projected that the combined soybean seed content of protein and oil should be increased by 10% by 2025 (http://unitedsoybean.org/about-usb/strategic-planning/). Most of the US soybeans have 59–62% combined protein and oil content. It is a great challenge to develop soybeans with >70% combined protein and oil. In addition, there is a need to develop soybean germplasm with increased meal protein (48–50%) by improving amino acid balance (10% increase in methionine, cysteine, and threonine) and environmental stability without reducing the seed oil or yield. Therefore, it is essential to identify and validate independent novel QTL for high protein content and to breakdown the negative correlation with yield and oil content. To meet this challenge, it is necessary to bridge the gap between biological and computational system by integrating multiple “omics” approaches, statistical genetic models, and bioinformatics tools, such as data pipelines, cloud computing, and user-friendly script developments. Despite the challenges, advances in “omics” technologies offers a promising potential to create next-generation soybeans with the desired seed composition traits. With constant developments in breeding technologies in conjunction with “omics” approaches, it is foreseeable in the future that a high yielding soybean cultivar with balanced amino acid, increased oil and protein content can be subsequently developed. In summary, this review provides a glimpse of advances made in improvement of soybean seed traits using integrated omics approaches. The discussed information would be a useful resource to accelerate desired seed composition improvement and, in turn, meet the global soybean demand in coming years.

### Conflict of interest statement

The authors declare that the research was conducted in the absence of any commercial or financial relationships that could be construed as a potential conflict of interest.

## Abbreviations

SSR: Simple Sequence Repeats
SNP: Single Nucleotide Polymorphism
WGS: Whole Genome Sequencing
WGRS: Whole Genome Re-Sequencing
GWAS: Genome-Wide Association Studies
GS: Genomic Selection
MAS: Marker-Assisted Selection
QTL: Quantitative Trait Loci
GBS: Genotyping-by-Sequencing
RIL: Recombinant Inbred Lines
Chr.: Chromosome.
