# Supplementary material for: Expanding Omics Resources for Improvement of Soybean Seed Composition Traits
Source: Front Plant Sci. 2015 Nov 24;6:1021. doi: 10.3389/fpls.2015.01021 (PMC4657443; doi:10.3389/fpls.2015.01021)
Supplement: Supplementary file 2 [file Table2.DOCX]

***Supplementary Material***

**Expanding omics resources for improvement of soybean seed composition traits**

**Juhi Chaudhary^1^, Gunvant Patil^1^, Humira Sonah^1,2^, Rupesh Deshmukh^1,2^, Tri D. Vuong^1^, Babu Valliyodan^1^ and Henry T. Nguyen^1^***

***Correspondence:**

Dr. Henry T. Nguyen [nguyenhenry@missouri.edu](mailto:nguyenhenry@missouri.edu)

**Supplementary Table 2:** Recent quantitative trait loci studies performed to identify genomic loci governing seed related traits in soybean.

| **Traits** | **Parents** | **Population size** | **Generation** | **QTL/SSR/SNP** | **Reference** |
| --- | --- | --- | --- | --- | --- |
| Seed coat cracking (SCC) | Keunolkong x Sinpaldalkong | 117 | F_11_ | Identified 16 loci associated with seed coat cracking | ([Ha et al., 2012](#_ENREF_2)) |
| 100 seed weight | Hefeng 25 x Dongnong L-5 | 125 | F_5:8_ | Identified 23 unconditional QTL and 17 conditional QTL | ([Han et al., 2012](#_ENREF_3)) |
| Seed coat wrinkling | PI 567743 x PI 87623 (Pop1),  T-311 x PI 87623 (Pop2),  PI 87623 x T-311 (Pop3) | Pop1: 175  Pop2: 066  Pop3:150 | F_2_ | Identified significant locus associated with wrinkling on Chr. 5 | ([Kebede et al., 2013](#_ENREF_5)) |
| Seed protein and oil content and seed weight  Seed isoflavones | Magellan x PI 438489B (Pop 1), Magellan x PI 567516C  Essex x William 82 | Pop 1: 216  Pop 2: 156  274 | F_5_  F_5:6_ | Identified 2 common QTL for oil and protein on Chr. 5 & 6; 1 QTL for seed weight on Chr. 6  Identified 21 QTL: 7 for genistein, 5 for daidzein, 3 for glycitein, and 6 for total isoflavone content | ([Pathan et al., 2013](#_ENREF_8))  ([Smallwood et al., 2014](#_ENREF_9)) |
| Seed size (length, width, 100 seed weight, and thickness) | Lishuizhongzihuang x Nannong493-1 | 504 | F_7:8_ | Identified two markers associated with seed traits | ([Xie et al., 2014](#_ENREF_13)) |
| Total isoflavone | Xiaoheidou x GR8836 | 184 | F_2:10_ | Identified 21 QTL on Chr. 8 | ([Zhang et al., 2014](#_ENREF_16)) |
| Seed storability | R18500 x MJ0004-6 | 129 | F_2:3_ | seed storability associated with 13 markers from six linkage groups | ([Dargahi et al., 2014](#_ENREF_1)) |
| Seed coat impermeability | PI 587982A x PI 594619 (Pop1),  PI 594619 x PI 587982A (Pop2), | Pop1: 199  Pop2: 052 | F_2_, F_2:3_ | Identified a single dominant gene that conditions seed coat impermeability in soybean PI 594619 | ([Kebede et al., 2014](#_ENREF_6)) |
| Seed calcium content | PI 407818 B x KS4303sp (Pop1),  PI 407818 B x PI 408052C (Pop2) | Pop1:120  Pop2:124 | F_2:4_ | Identified new calcium QTL and markers on Chr. 18 | ([Orazaly et al., 2014](#_ENREF_7)) |
| Seed sucrose content | MFS-553 x PI 243545 | 220 | F_2:3,_ F_3:4,_ F_3:5_ | Three novel QTL located on Chr.5, 9, and 16 for sucrose content were identified | ([Zeng et al., 2014](#_ENREF_14)) |
| Seed weight | Ohsuzu x Athow (PI 595926) (Pop1),  PI 593654 x PI 561396 (Pop2) | Pop1:225  Pop2:250 | F_6_ | One QTL positioned between Sat_284 and Sat_292 on Chr. 17 was identified | ([Kato et al., 2014](#_ENREF_4)) |
| Soluble sugar content | V97-3000 x V99-5089 | 170 | F_2:3_ | A total of 11 QTL detected for sugar content: one for glucose, three each for fructose and sucrose, and two each for raffinose and stachyose | ([Wang et al., 2014](#_ENREF_11)) |
| Seed protein content | R05-1415 x R05-638 (Pop1),  V97-1346 x R05-4256 (Pop2), | Pop1:242  Pop:214 | F_2_ | One major protein QTL on Chr. 20, 4 QTL on Chr. 1, 5, and 14 | ([Wang et al., 2015](#_ENREF_10)) |
| Stachyose content | Osage x V99-5089 | 129 | F_2:3_ | A major QTL on Chr. 11 and a novel QTL on Chr. 10 for stachyose content were identified | ([Zeng et al., 2015](#_ENREF_15)) |
| Protein and Amino Acid | PI595645 x PI619083 | 140 | F5 derived | Chr. 14, 15, 17, and 20 associated with protein and amino acid | ([Warrington et al., 2015](#_ENREF_12)) |

DTF: days from planting to flowering; DTM: days from planting to maturity; PN: pod number; HSW: 100 seed weight; YLD: yield per plant

**References**

Dargahi, H., Tanya, P., and Srinives, P. (2014). Mapping of the genomic regions controlling seed storability in soybean (Glycine max L.). *Journal of genetics* 93**,** 365-370.

Ha, B.-K., Kim, H.-K., and Kang, S.-T. (2012). Mapping QTLs with epistatic effects and QTL-by-environment interactions for seed coat cracking in soybeans. *Euphytica* 186**,** 933-942.

Han, Y., Xie, D., Teng, W., Sun, J., and Li, W. (2012). QTL underlying developmental behaviour of 100‐seed weight of soybean. *Plant Breeding* 131**,** 600-606.

Kato, S., Sayama, T., Fujii, K., Yumoto, S., Kono, Y., Hwang, T.-Y., Kikuchi, A., Takada, Y., Tanaka, Y., and Shiraiwa, T. (2014). A major and stable QTL associated with seed weight in soybean across multiple environments and genetic backgrounds. *Theor. Appl. Genet.* 127**,** 1365-1374.

Kebede, H., Smith, J.R., and Ray, J.D. (2013). A new gene that controls seed coat wrinkling in soybean. *Euphytica* 189**,** 309-320.

Kebede, H., Smith, J.R., and Ray, J.D. (2014). Identification of a single gene for seed coat impermeability in soybean PI 594619. *Theor. Appl. Genet.* 127**,** 1991-2003.

Orazaly, M., Chen, P., Zhang, B., and Zeng, A. (2014). Quantitative trait loci mapping for seed calcium content of soybean. *Crop Sci.* 54**,** 500-506.

Pathan, S.M., Vuong, T., Clark, K., Lee, J.-D., Shannon, J.G., Roberts, C.A., Ellersieck, M.R., Burton, J.W., Cregan, P.B., and Hyten, D.L. (2013). Genetic mapping and confirmation of quantitative trait loci for seed protein and oil contents and seed weight in soybean. *Crop Sci.* 53**,** 765-774.

Smallwood, C.J., Nyinyi, C.N., Kopsell, D.A., Sams, C.E., West, D.R., Chen, P., Kantartzi, S.K., Cregan, P.B., Hyten, D.L., and Pantalone, V.R. (2014). Detection and Confirmation of Quantitative Trait Loci for Soybean Seed Isoflavones. *Crop Sci.* 54**,** 595-606.

Wang, J., Chen, P., Wang, D., Shannon, G., Zeng, A., Orazaly, M., and Wu, C. (2015). Identification and mapping of stable QTL for protein content in soybean seeds. *Mol. Breeding* 35**,** 1-10.

Wang, Y.Q., Chen, P.Y., and Zhang, B. (2014). Quantitative trait loci analysis of soluble sugar contents in soybean. *Plant Breeding* 133**,** 493-498. doi: 10.1111/pbr.12178.

Warrington, C., Abdel-Haleem, H., Hyten, D., Cregan, P., Orf, J., Killam, A., Bajjalieh, N., Li, Z., and Boerma, H. (2015). QTL for seed protein and amino acids in the Benning× Danbaekkong soybean population. *Theor. Appl. Genet.* 128**,** 839-850.

Xie, F.-T., Niu, Y., Zhang, J., Bu, S.-H., Zhang, H.-Z., Geng, Q.-C., Feng, J.-Y., and Zhang, Y.-M. (2014). Fine mapping of quantitative trait loci for seed size traits in soybean. *Mol. Breeding* 34**,** 2165-2178.

Zeng, A., Chen, P., Shi, A., Wang, D., Zhang, B., Orazaly, M., Florez-Palacios, L., Brye, K., Song, Q., and Cregan, P. (2014). Identification of Quantitative Trait Loci for Sucrose Content in Soybean Seed. *Crop Sci.* 54**,** 554-564.

Zeng, A., Chen, P., Zhang, B., Orazaly, M., Florez‐Palacios, L., and Brye, K.R. (2015). Identification and confirmation of quantitative trait loci for stachyose content in soybean seed. *Plant Breeding* 134**,** 178-185.

Zhang, H.J., Li, J.W., Liu, Y.J., Jiang, W.Z., Du, X.L., Li, L., Li, X.W., Su, L.T., Wang, Q.Y., and Wang, Y. (2014). Quantitative trait loci analysis of individual and total isoflavone contents in soybean seeds. *Journal of genetics* 93**,** 331-338.
