# Supplementary material for: Expanding Omics Resources for Improvement of Soybean Seed Composition Traits
Source: Front Plant Sci. 2015 Nov 24;6:1021. doi: 10.3389/fpls.2015.01021 (PMC4657443; doi:10.3389/fpls.2015.01021)
Supplement: Supplementary file 3 [file Table3.DOCX]

***Supplementary Material***

**Expanding omics resources for improvement of soybean seed composition traits**

**Juhi Chaudhary^1^, Gunvant Patil^1^, Humira Sonah^1,2^, Rupesh Deshmukh^1,2^, Tri D. Vuong^1^, Babu Valliyodan^1^ and Henry T. Nguyen^1^***

***Correspondence:**

Dr. Henry T. Nguyen [nguyenhenry@missouri.edu](mailto:nguyenhenry@missouri.edu)

**Supplementary Table 3:** Summary of significant genome-wide association mapping studies performed in soybean

| **Trait** | **Platform/Approach** | **Summary** | **Reference** |
| --- | --- | --- | --- |
| **Seed related Traits** | | | |
| Pod and seed number, seed weight and seed yield | 1,536 SNP chips | 19 SNPs and 5 haplotypes associated with soybean yield and yield components | ([Hao et al., 2012b](#_ENREF_3)) |
| Oil and protein | Illumina Infinium and GoldenGate Assays | 40 SNPs in 17 genomic regions associated with seed protein; 25 SNPs in 13 regions associated with seed oil | ([Hwang et al., 2014](#_ENREF_4)) |
| Protein, oil, amino acids, sucrose and stachyose | SoySNP50K iSelect BeadChips (Song et al., 2013) | Identified candidate loci and SNP associated with essential amino acid content | ([Vaughn et al., 2014](#_ENREF_7)) |
| Domestication and improvement traits (oil content, seed weight) | Re-sequencing | Identified several GWAS signals associated with seed related and other domestication traits. | ([Zhou et al., 2015b](#_ENREF_11)) |
| Seed weight, oil, protein and domestication traits | Genotyping by sequencing (GBS) | Demonstrated the integration of GBS and GWAS and identified several loci associated with oil, protein and other traits. | ([Sonah et al., 2014](#_ENREF_6)) |
| Flowering time and seed size | Restriction Associated DNA-Sequencing (RAD-seq) | Identified a total of 48 loci associated with domestication related traits. 4 QTL were associated with flowering time and 8 loci for seed size | ([Zhou et al., 2015a](#_ENREF_10)) |
| **Other Traits** | | | |
| Iron deficiency | Illumina GoldenGate assay | 9 loci from seven genomic locations associated with iron deficiency were identified | ([Mamidi et al., 2011](#_ENREF_5)) |
| Chlorophyll and chlorophyll fluorescence parameters | Illumina GoldenGate Assay | 51 SNPs associated with chlorophyll and chlorophyll fluorescence parameter were identified | ([Hao et al., 2012a](#_ENREF_2)) |
| Water use efficiency | SoySNP50K iSelect BeadChips (Song et al., 2013) | 39 SNPs associated with 21 different loci for carbon isotope ratio. | ([Dhanapal et al., 2015](#_ENREF_1)) |
| Sudden death syndrome | Illumina SoySNP50K iSelect BeadChip and Illumina SoySNP6k iSelect BeadChip | 7 loci in correlation with previously mapped loci and 13 novel loci for SDS resistance | ([Wen et al., 2014](#_ENREF_8)) |
| Flowering time, maturity dates and plant height | SoySNP50K iSelect BeadChips (Song et al., 2013) | 27, 6, 18 and 27 loci for days to flowering, maturity, duration of flowering-to-maturity and plant height, respectively were identified | ([Zhang et al., 2015](#_ENREF_9)) |

**References**

Dhanapal, A.P., Ray, J.D., Singh, S.K., Hoyos-Villegas, V., Smith, J.R., Purcell, L.C., King, C.A., Cregan, P.B., Song, Q., and Fritschi, F.B. (2015). Genome-wide association study (GWAS) of carbon isotope ratio (δ13C) in diverse soybean [Glycine max (L.) Merr.] genotypes. *Theor. Appl. Genet.* 128**,** 73-91.

Hao, D., Chao, M., Yin, Z., and Yu, D. (2012a). Genome-wide association analysis detecting significant single nucleotide polymorphisms for chlorophyll and chlorophyll fluorescence parameters in soybean (Glycine max) landraces. *Euphytica* 186**,** 919-931.

Hao, D., Cheng, H., Yin, Z., Cui, S., Zhang, D., Wang, H., and Yu, D. (2012b). Identification of single nucleotide polymorphisms and haplotypes associated with yield and yield components in soybean (Glycine max) landraces across multiple environments. *Theor. Appl. Genet.* 124**,** 447-458.

Hwang, E.-Y., Song, Q., Jia, G., Specht, J.E., Hyten, D.L., Costa, J., and Cregan, P.B. (2014). A genome-wide association study of seed protein and oil content in soybean. *BMC Genomics* 15**,** 1.

Mamidi, S., Chikara, S., Goos, R.J., Hyten, D.L., Annam, D., Moghaddam, S.M., Lee, R.K., Cregan, P.B., and Mcclean, P.E. (2011). Genome-wide association analysis identifies candidate genes associated with iron deficiency chlorosis in soybean. *Plant Genome* 4**,** 154-164.

Sonah, H., O'donoughue, L., Cober, E., Rajcan, I., and Belzile, F. (2014). Identification of loci governing eight agronomic traits using a GBS‐GWAS approach and validation by QTL mapping in soya bean. *Plant Biotech. J.*

Vaughn, J.N., Nelson, R.L., Song, Q., Cregan, P.B., and Li, Z. (2014). The Genetic Architecture of Seed Composition in Soybean Is Refined by Genome-Wide Association Scans Across Multiple Populations. *G3* 4**,** 2283-2294.

Wen, Z., Tan, R., Yuan, J., Bales, C., Du, W., Zhang, S., Chilvers, M.I., Schmidt, C., Song, Q., and Cregan, P.B. (2014). Genome-wide association mapping of quantitative resistance to sudden death syndrome in soybean. *BMC genomics* 15**,** 809.

Zhang, J., Song, Q., Cregan, P.B., Nelson, R.L., Wang, X., Wu, J., and Jiang, G.-L. (2015). Genome-wide association study for flowering time, maturity dates and plant height in early maturing soybean (Glycine max) germplasm. *BMC genomics* 16**,** 217.

Zhou, L., Wang, S.-B., Jian, J., Geng, Q.-C., Wen, J., Song, Q., Wu, Z., Li, G.-J., Liu, Y.-Q., and Dunwell, J.M. (2015a). Identification of domestication-related loci associated with flowering time and seed size in soybean with the RAD-seq genotyping method. *Sci. Rep.* 5.

Zhou, Z., Jiang, Y., Wang, Z., Gou, Z., Lyu, J., Li, W., Yu, Y., Shu, L., Zhao, Y., and Ma, Y. (2015b). Resequencing 302 wild and cultivated accessions identifies genes related to domestication and improvement in soybean. *Nat. Biotechnol.*
