# Supplementary material for: Expanding Omics Resources for Improvement of Soybean Seed Composition Traits
Source: Front Plant Sci. 2015 Nov 24;6:1021. doi: 10.3389/fpls.2015.01021 (PMC4657443; doi:10.3389/fpls.2015.01021)

Supplementary Material

Expanding omics resources for improvement of soybean seed composition traits

Juhi Chaudhary[1], Gunvant Patil[1], Humira Sonah[1,2], Rupesh Deshmukh[1,2], Tri D. Vuong[1], Babu Valliyodan[1] and Henry T. Nguyen[1]*

***Correspondence:**
Dr. Henry T. Nguyen nguyenhenry@missouri.edu

Supplementary Figure 1: Number of published articles reporting the uses of 'omics' tools in soybean. The data was accessed from PubMed (http://www.ncbi.nlm.nih.gov/pubmed) by searching 'genome' or genomics', 'transcriptome' or 'transcriptomic', and 'proteome'or 'proteomic' AND 'soybean' in title or abstract on 15 March 2015.

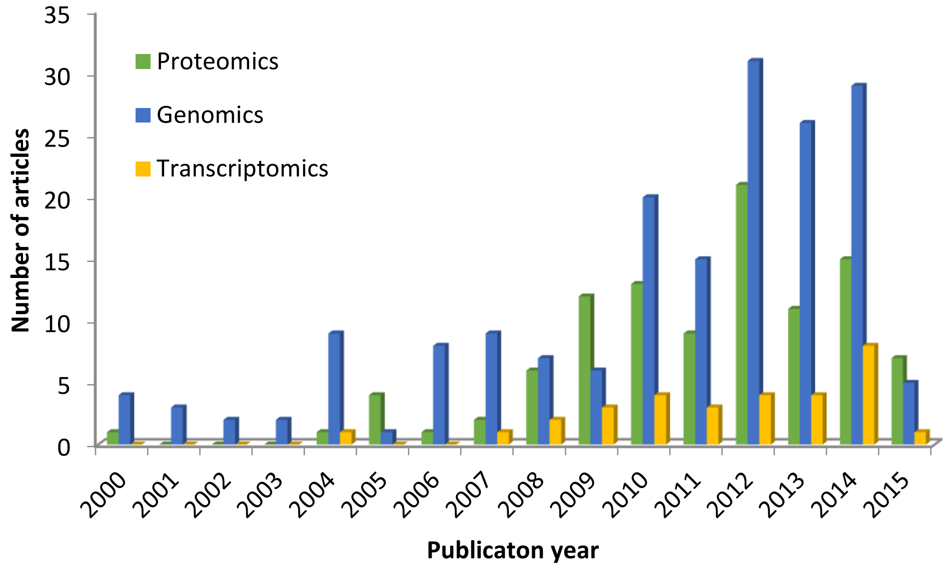

Supplement: Supplementary file 4 [file Image1.PDF]
