# Supplementary material for: Expanding Omics Resources for Improvement of Soybean Seed Composition Traits
Source: Front Plant Sci. 2015 Nov 24;6:1021. doi: 10.3389/fpls.2015.01021 (PMC4657443; doi:10.3389/fpls.2015.01021)
Supplement: Supplementary file 5 [file Image2.PDF]

Supplementary Material

Expanding omics resources for improvement of soybean seed composition traits

Juhi Chaudhary[1], Gunvant Patil[1], Humira Sonah[1,2], Rupesh Deshmukh[1,2], Tri D. Vuong[1], Babu Valliyodan[1] and Henry T. Nguyen[1]*

***Correspondence:**
Dr. Henry T. Nguyen nguyenhenry@missouri.edu

Supplementary Figure 2: Distribution of quantitative trait loci studies focusing different seed related traits in soybean. The data was collected from Web of Science database (http://apps.webofknowledge.com/UA_GeneralSearch_input.do?product=UA&search_mode=GeneralSearch&SID=1CDXnXNwLD3N15okVxU&preferencesSaved=) with keyword in title 'soybean' or 'Glycine' and 'QTL and map' in topic field on 15 March 2015.

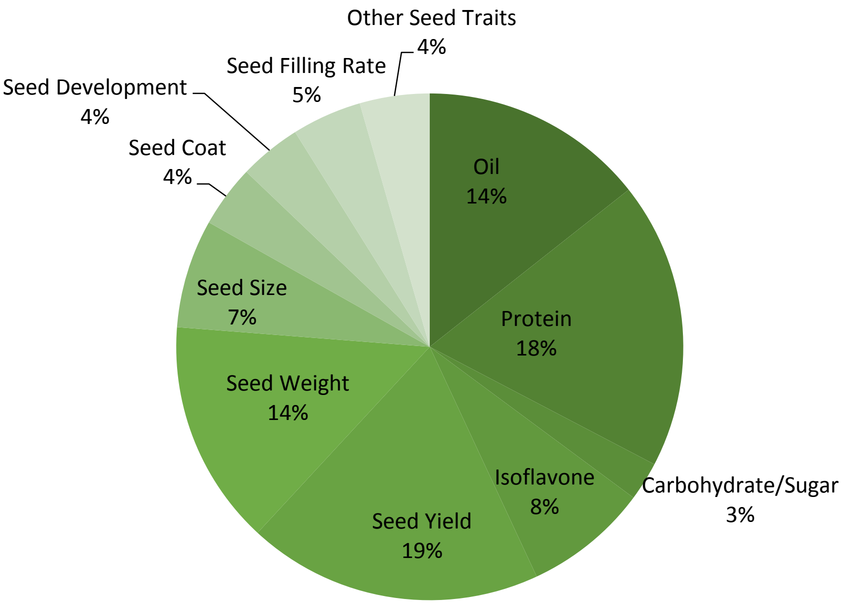

No. of Seed related QTL Papers published
