# Supplementary material for: Expanding Omics Resources for Improvement of Soybean Seed Composition Traits
Source: Front Plant Sci. 2015 Nov 24;6:1021. doi: 10.3389/fpls.2015.01021 (PMC4657443; doi:10.3389/fpls.2015.01021)

Supplementary Material

Expanding omics resources for improvement of soybean seed composition traits

Juhi Chaudhary[1], Gunvant Patil[1], Humira Sonah[1,2], Rupesh Deshmukh[1,2], Tri D. Vuong[1], Babu Valliyodan[1] and Henry T. Nguyen[1]*

***Correspondence:**
Dr. Henry T. Nguyen nguyenhenry@missouri.edu

Supplementary Figure 3: Number of soybean gene expression datasets available in Gene Expression Omnibus (GEO) in the past few years. Data was collected from (http://www.ncbi.nlm.nih.gov/gds) on 30 March 2015. A total of 1,629 studies was found on manual inspection for soybean seed traits.

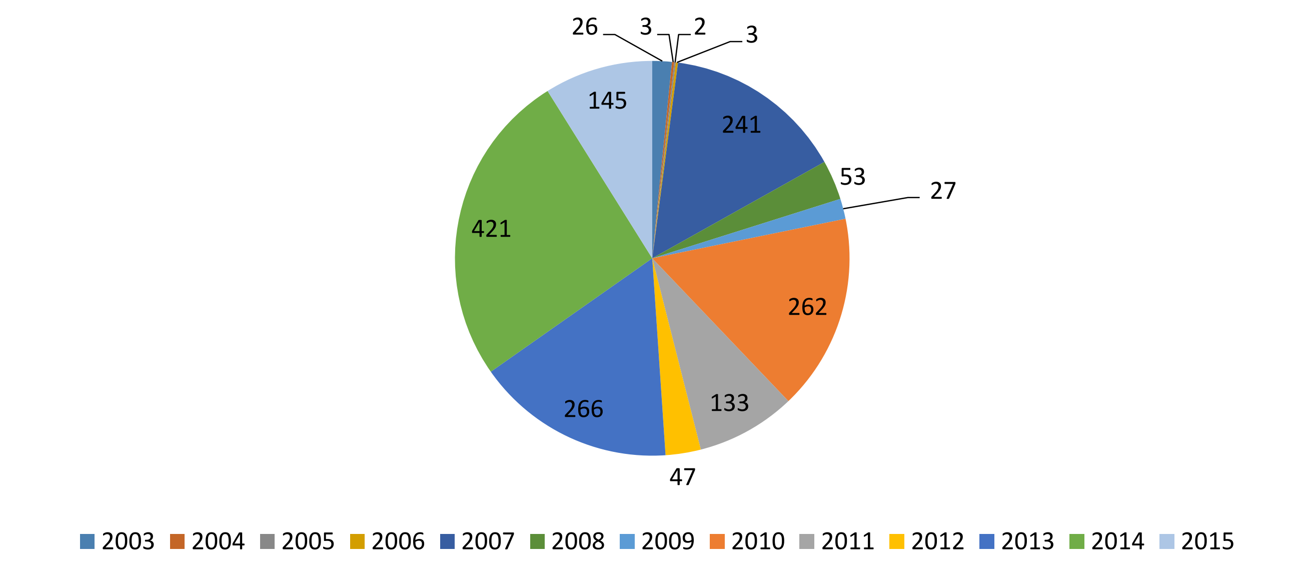

Supplement: Supplementary file 6 [file Image3.PDF]
